# Regrets Associated with Providing Healthcare: Qualitative Study of Experiences of Hospital-Based Physicians and Nurses

**DOI:** 10.1371/journal.pone.0023138

**Published:** 2011-08-02

**Authors:** Delphine S. Courvoisier, Thomas Agoritsas, Thomas V. Perneger, Ralph E. Schmidt, Stéphane Cullati

**Affiliations:** 1 Division of Clinical Epidemiology, University Hospitals of Geneva, Geneva, Switzerland; 2 Faculty of Medicine, University of Geneva, Geneva, Switzerland; 3 Division of General Internal Medicine, University Hospitals of Geneva, Geneva, Switzerland; 4 Swiss Center for Affective Sciences, University of Geneva, Geneva, Switzerland; 5 Quality of Care Service, University Hospitals of Geneva, Geneva, Switzerland; 6 Institute of Demographic and Life Course Studies, University of Geneva, Geneva, Switzerland; Charité, Campus Benjamin Franklin, Germany

## Abstract

**Background:**

Regret is an unavoidable corollary of clinical practice. Physicians and nurses perform countless clinical decisions and actions, in a context characterised by time pressure, information overload, complexity and uncertainty.

**Objective:**

To explore feelings associated with regretted clinical decisions or interventions of hospital-based physicians and nurses and to examine how these regrets are coped with.

**Method:**

Qualitative study of a volunteer sample of 12 physicians and 13 nurses from Swiss University Hospitals using semi-structured interviews and thematic analysis

**Results:**

All interviewees reported at least one intense regret, which sometimes led to sleep problems, or taking sickness leave. Respondents also reported an accumulation effect of small and large regrets, which sometimes led to quitting one's unit or choosing another specialty. Respondents used diverse ways of coping with regrets, including changing their practices and seeking support from peers and family but also suppression of thoughts related to the situation and ruminations on the situation. Another coping strategy was acceptance of one's limits and of medicine's limits. Physicians reported that they avoided sharing with close colleagues because they felt they could lose their credibility.

**Conclusions:**

Since regret seems related to both positive and negative consequences, it is important to learn more about regret coping among healthcare providers and to determine whether training in coping strategies could help reduce negative consequences such as sleep problems, absenteeism, or turnover.

## Introduction

Regret is an unavoidable corollary of clinical practice. Physicians and nurses perform countless clinical decisions and actions during their workday, in a context characterised by time pressure, information overload, complexity and uncertainty [1]. Inevitably, some of these decisions and actions will be suboptimal or wrong, or appear such to their authors in retrospect. Indeed, a worsening of patients' condition may follow even the best decisions and actions. Regret is an emotion experienced when one believes that the current situation would be better if one had behaved differently [2]. Given that 98% of interns have admitted to committing medical errors [3], regret is presumably frequent among healthcare professionals. Little is known about the experience of regret in physicians and nurses.

Furthermore, those who experience regret usually rely on coping strategies. One classification of these strategies proposes three categories: cognitive, action-oriented, and social [2]. Cognitive strategies include suppression of regret-related thoughts. Action-oriented strategies aim at preventing similar situations in the future. Social strategies consist in seeking either an attentive ear or concrete support from others. How these strategies are used by physicians and nurses, and how effective they are, is poorly understood.

Why should we study regret in health care? First, regret may influence clinical decision-making. While the influence of cognitive processes on medical decisions has been extensively studied [4], [5], the impact of affective processes has as yet received little attention [6], [7]. Regret plays a key role in various types of decisions [8], [9], including medical decisions [10], [11], [12]. For instance, prior to medical decision, physicians' intentions to vaccinate adolescent girls against human papillomavirus are correlated with anticipated regret of inaction [11]. The level of acceptable regret (i.e., regret that a decision maker finds acceptable upon making a wrong decision) influence the decision to order a test [12]. Second, regret also influences physical and mental health. The intensity of regret is associated with feelings of loss [13] and with a wide range of physical and psychological symptoms [14]. Regret may also disturb sleep [15]. Given that sleep loss may lead to attention deficits [16] and thus to an increased risk of errors, a vicious circle between errors, regrets, and insomnia could thus emerge [17]. Finally, intense regrets may decrease job satisfaction and may eventually lead to the decision to change jobs.

The aim of this study is to explore feelings of regret associated with clinical decisions or actions of hospital-based physicians and nurses, and to investigate how healthcare professionals cope with these feelings. As healthcare professionals have to make many decisions and action during their workday, an additional aim of this study is to examine whether the theoretical framework used to describe coping strategies is consistent with physicians' and nurses' feelings of regret and coping strategies.

## Methods

The study was approved by the Research Ethics Committee of the University Hospitals of Geneva. In this qualitative study, as accepted by the Research Ethics Committee, verbal informed consent was obtained from participants at the beginning of the tape-recorded interview. The names of the interviewees were not evoked during the tape-recorded interview and thus the names did not appear in the transcripts. Thus, only the interviewer (SC) knew the identity of the participants.

### Participants and setting

Over five months, 25 participants were recruited by posting information about the study on the intranet news page of the University Hospitals of Geneva and on the website of the clinical epidemiology division, as well as by presenting the study during division seminars. We sought to include similar numbers of physicians and nurses, representing all types of clinical practice. Twelve participants were physicians (4 female; 5 senior physicians) and 13 were nurses (11 female; 3 senior nurses). Participants came from departments of anaesthesiology (N = 6), internal medicine (N = 4), visceral surgery (N = 3), obstetrics (N = 2), infectious diseases (N = 2), paediatrics (N = 1), emergency psychiatry (N = 1), intensive care (N = 1), haematology-oncology (N = 1), nursing management (N = 1), international and humanitarian medicine (N = 1), and palliative care (N = 1). Mean age was 40 years (range 27–64).

### Interviews

Face-to-face semi-structured interviews were conducted between April 2009 and September 2009 (questions: Box S1). At the beginning of the interview, regret was defined by the interviewer and clearly differentiated from medical error (i.e., medical error could elicit regret but regret could stem from situations that did not involve a medical error). Interviewees were then asked to describe at least two patient-care situations in which they experienced regret, one in which they felt they made a mistake and one in which they did not. Specific questions concerned the situation eliciting regret, the emotions, thoughts and physical sensations occurring during the situation, the consequences for the healthcare professional's life, and the coping strategies used by the healthcare professional (Box S1). The interview included two closed-format items: respondents rated the intensity of their regrets on a visual analogue scale from 0 (no regret) to 10 (very high), at the time of occurrence of the event, and at the time of the interview. Interviews lasted on average 52 minutes (range 31–88). All interviews were tape-recorded and fully transcribed.

### Analysis

Analysis focused on identifying thoughts and other emotions experienced in relation to situations in which respondents experienced regret, and on identifying coping strategies used by respondents in these situations. SC and DC read the first 10 interviews independently, noting significant emergent themes (e.g., use of acceptance of one's limits to cope with regret) and passages. Interviews were also examined for instances of pre-defined, literature-based codes related to coping strategies (e.g., use of suppression coping strategy) and for a documented consequence of regret: sleep difficulties. After comparing their results and resolving any discrepancies, a definitive list of codes and a set of coding rules were developed. SC then coded all interviews, which were reviewed by DC. Transcripts were also reviewed by physicians to provide an accurate interpretation of the clinical situations. Coding was performed using Nvivo 8 software (QSR International Pty Ltd; Doncaster, Victoria, Australia). Approximately 75% of the theory-driven coding categories were very rarely used and therefore discarded. In the end, half the categories used were predetermined and half were emergent.

## Results

The 25 participants reported 61 situations in which they experienced regrets. Three situations were excluded because they did not concern patient care but relationships with colleagues, and another was excluded because the respondent was not able to describe a specific situation. Finally, 57 regretted situations were included in the analysis. On average, the respondents reported two personally significant regrets (range 1 to 5).

Respondents were often very emotional during the interviews and seven out of 25 cried or were on the verge of tears. Furthermore, several respondents were very interested in any help we could provide in dealing with regrets.

### Regret-inducing situations

Regret was experienced in a wide range of situations including diagnosis, administration of a treatment and its outcomes, patient healthcare management and inter-personal relations between the patient (or his family) and the healthcare provider. Additionally, several nurses reported situations in which the points of view of the patient and of the physician differed; these situations related either to different conceptions about end-of-life care (palliative care vs. pursuing curative interventions), or physicians' respect of the patient's intimacy.

*“I am cleaning a patient, the drapes are closed, the physician arrives, I hear noises, he enters without asking and voluntarily, I say: ‘Careful, we're doing intimate cleaning’ […]. I shock on purpose, some are embarrassed, and others don't even hear me.”* (nurse)

Most situations took place in the hospital, but some respondents depicted other settings such as homecare or occupational health. Only half of the respondents reported a situation in which the regret was linked to a mistake or error (examples: Box S2). Respondents also regretted situations in which no obvious mistakes were committed (examples: Box S3). Indeed, three fourth of the reported situations were not related to a mistake. Each citation will be preceded by a number in bracket indicating which situation the citation refers to or by an X if the citation is a generality.

Many respondents described intense experiences that dated back to their first years of clinical practice. Strong regrets typically entailed a great shock to professional identity, for both physicians and nurses.

[X] *“A questioning of my professional abilities. Very soon, I told myself that people wouldn't trust me anymore, I really questioned myself.”* (nurse)

Additionally, more than half of the respondents reported that they regularly experienced regrets for “small things” that did not have major consequences.

[X] *“I'd say one event erases another. When I had other worries afterward, we can't deal with everything at the same time […] We have other reasons to have regrets and then the first regrets lose their intensity (laughs).”* (physician)

[X] *“Finally, I feel that we could almost have regrets every day, be they small or large.”* (nurse)

Usually these daily minor regrets were quickly forgotten, but there was an accumulation effect.

[X] *“It's true that we have regrets everyday in a way, not vital stuff but for example, we make a great deal of effort to take care of the patients and then we don't have time to see them again […]. We have kind of small regrets every day that finally lead to small frustrations about our work.”* (physician)

#### Regret intensity

On the visual analogue scale from 0 (no regret) to 10 (very high), mean intensity at the time of the event was 7 [range 1.5 to 10; N = 52] and 3 at the time of the interview [range 0 to 10; N = 25]. Intensity was equivalent for regrets that were related to a mistake and for regrets that weren't.

#### Emotions

Generally, respondents expressed more than one other emotion felt during the regretted situations. In decreasing frequency, these emotions were guilt, anger, sadness, shame, helplessness, and a feeling of unfairness/injustice. Anger was either self-oriented or oriented against a target (e.g., the patient, the institution, the situation, colleagues).

[2.1] *“A very strong feeling of shame. Shame, yes, very strong. And then, in a way, of anger […] towards myself, and towards the institution too.”* (physician).

[3.10] *“I find it a pity, as a human being, that he left (died) like that. Especially the discomfort also, not knowing how he left. We found him with the mask between his legs… Did he struggle, did he… It's sadness yes… (long pause).”* (nurse)

*Thoughts*

The majority of regrets were related to an action, the remainder were regrets of inaction. During the situation, respondents either thought about the consequences of their action or about what else they could have done:

[3.4] *“With the fear, where I tell myself ‘if I make a mistake, if anything happens…’”* (physician)

[2.1] *“Why didn't I go explain myself to the patient, why didn't I apologize?”* (physician).

#### Physical manifestations

During the regretted situation, more than half of the respondents also experienced physical symptoms, such as, in decreasing frequency, stomach ache, throat/chest oppression, headache, hot flushes, trembling, palpitations.

[2.8] *“And there, I thought I was going to faint, I felt so bad (laughs) […] My heart was beating at 200. […] heart burn, feelings of squeezing, of tingling […] And then every time we talked about it, there was a lump [in my throat].”* (nurse)

[2.6] *“It's more the heart that goes too fast, the impression that the floor vanishes under my legs […] and hot flushes, maybe even feeling dizzy.”* (nurse)

### Regret regulation strategies

When asked how they coped with their regrets, both physicians and nurses reported using several strategies, which were grouped under three headings: cognitive, action-oriented and/or social strategies.

#### Cognitive strategies

Respondents described four main cognitive strategies. The most frequent strategies were rumination (i.e., recurrent thoughts about the situation) and suppression (i.e., trying to not think about what happened). Sometimes, suppression was considered as a transitory measure, a voluntary way to block overwhelming emotions.

[2.11] *“And then […] I avoided it, I didn't think about it anymore, I didn't think about it anymore and then it's when I went back to work that it all came back, because I thought about it again”* (nurse)

[2.10] *“I blocked it for a while but then it came back, it's this image of this patient who took off her (mask), who was all grey, well, she didn't have enough oxygen.”* (nurse).

[3.8] *“It's something that we feel for a while and then later, well, we try to evacuate it quite quickly because it's useless anyway.”* (physician)

Furthermore, suppression was often related to rumination: respondents who tried not to think about what happened often involuntarily thought about it even more when nothing distracted them, for example just before sleep.

[3.5] *“In this type of situation, we redo the scenario 50 times in our head, again and again, we think, ‘I should have done this differently’.”* (physician)

[3.12] *“Even at night, well, you don't sleep, you take a sleeping pill because at 1 am, 2 am, well […] it doesn't stop when you leave the hospital, no it's quite hard, quite difficult.”* (nurse)

The third most frequent strategy was acceptance of both one's inability to do the right thing or to help the patient and/or acceptance of the limitations of medicine itself.

[3.14] *“It helps also to tell yourself that you did what you could, we're just human after all, there is a moment when it's not possible [to keep going] anymore, it's not possible whatever happens.”* (nurse)

[X] *“And everybody knows that we make mistakes in our lives and maybe once it cost the life of a patient.”* (physician)

[3.7] *“I didn't have enough distance towards nature and towards the acceptance that death happens even for a young (adult) if he has a severe accident.”* (physician)

Finally, regret sometimes led to self-attacking (i.e., criticise one's whole self and not only one's action).

[2.6] *“So in this situation, for me, it was very strong, at this moment, I told myself that I wasn't good for anything anymore.”* (nurse)

[3.4] *“It's a regret towards myself and also towards the patient, and I thought I'm really an idiot.”* (physician)

#### Action-oriented strategies

Healthcare professionals who implemented action-oriented strategies mostly did so after intense regrets. Three main action-oriented strategies were reported. First, respondents often tried to take responsibility for the event (e.g., a nurse bathed the dead patient that she felt she had not supported as much as she should have, physicians went to talk to the patient to give explanations or present excuses). Second, some healthcare professionals reported that they became more vigilant about this type of event and tended to repeat check-up procedures more often.

[X] *“After any mistake that I make, every time, I call myself into question and […] I organise things, actions so that the risk is decreased.”* (nurse)

[2.8] *“For sure I check three times rather than twice the name, the dose, the patient, the identity bracelet, the name on the bed.”* (nurse).

Finally, guidelines were sometimes created to avoid similar incidents in the future.

#### Social strategies

Social strategies were regularly adopted by nearly all interviewees. Social support was most often sought among peers (i.e., colleagues of the same profession), but sometimes also by talking to one's relatives (e.g., spouse).

[3.14] *“In private life, sometimes, I could talk about it 4, 5, 10 times during the week if it was necessary until it dried out and a new dramatic event happened (laughs) and I focused on something else.”* (nurse).

[2.4] *“I told my wife […] I can really talk freely to her without anyone saying, well you're an idiot, we listen to each other and after that, it's all good.”* (physician).

While respondents often reported that they used social support to reduce negative outcomes such as rumination (2.7) and urge to quit their job (3.11), they also underlined the limits of this strategy (3.13).

[2.7] *“Because what helped me, and what put this situation out of my head, it's that I could talk to my colleagues, ask their opinion, what they would have done differently, what strategies they would have used.”* (nurse)

[3.11] *“I think that with all these situations we encounter […] if there wasn't all this listening, the sharing and the support, I think we would all have left a long time ago.”* (nurse)

[3.13] *“I think that we need to talk. […] sometimes it just feels good […] I think it's also a way to release… […] it's like letting steam out, it doesn't help the situation, but we have the impression that is makes us feel better, it's better just talking about it between us.”* (nurse)

Physicians typically avoided sharing their regrets with peers of the same ward or service and preferentially turned to colleagues from other services. In contrast, nurses typically sought support from colleagues of the same service. Barriers to social strategies were expressed only by a few physicians, not by nurses.

[3.1] *“It's still a sign of weakness, and, shall we say, you're swimming with sharks.”* (physician)

[3.4] *“There, I knew there were no visible negative consequences [for the patient], whereas I had a fear that there could be negative consequences for me, […] I had no wish to go see my boss saying well I messed up, because that could have had negative consequences.”* (physician).

### Consequences

Almost all respondents indicated that the regrets they described influenced their private or professional life. The most frequent consequence was sleep difficulties (for about half). Respondents reported either thinking too much about the situation before sleeping, bad quality of sleep, and/or nightmares.

[2.1] *“A certain nervousness, you have nightmares, you don't sleep well.”* (physician)

[3.9] *“I was not thinking about it constantly, even though it came back, yes, mostly at night, going to sleep, that the situation turned in my head.”* (nurse).

Another consequence was loss of confidence.

[3.1] *“A big loss of confidence in my capacities, so you become a bit timid in your relations with the patients, in your decision making, with the nurses you feel bad.”* (physician),

[3.9] *“When I came back to work, I had this fear of not being good enough, you know, when self-esteem goes down, and then you don't feel like you're up to it.”* (nurse)

Respondents also reported concentration difficulties.

[2.1] *“‘I made a mistake, I made a mistake’ that came back and that made it harder to concentrate on the task at hand.”* (physician)

[2.6] *“I was at rope's end, physically and emotionally. So, I had to take a leave for two days.”* (nurse)

For some respondents, the regret-eliciting episodes set the course for their careers. Indeed, four participants (two nurses and two physicians) reported that the regret-inducing episode had led them to quit a service and/or to opt for another specialty.

[X] *“It's true that eventually it […] decided the orientation of my career, in the sense that, I don't know if it is this episode or another one […] I opted for a specialty […] in which errors happen often but which is also, let's say, a very structured specialty: we know the errors, we know the risks, and the safeguards are in place.”* (physician)

[X] *“It progressively made me leave this unit.”* (nurse)

## Discussion

All interviewees reported at least one intense regret, which often stemmed from situations that occurred during the first years of clinical practice. Even though half the respondents reported one error-related situation, most situations described were not related to errors. As expected from general population findings, both suppression and rumination of thoughts related to the regret-inducing situation were frequent [18]. This is consistent with the respondents' reports of sleep troubles as suppression is a known cause of such problems [18]. Furthermore, both physicians and nurses often sought social support, mainly from colleagues. Some examined the event inducing the regret and made changes in their practice, as advised by literature on coping with mistakes [19].

This study uncovered some new results that may have important consequences at several levels. For healthcare professionals, regret often led to loss of confidence and self-esteem. This may have a negative impact on the performance of clinical tasks. Furthermore, interviewees reported a cumulative effect of small regrets, that, separately, were soon forgotten, but, taken together, amounted to an important burden. This accumulation could be related to emotional exhaustion, one of the components of burnout [20], which in turn could lead to increased turnover [21], [22]. It could also be related to depression, which may result in higher rates of medications errors [23].

At the level of patient care, regret may reduce the risk of errors, if it motivated learning from one's mistakes [24] and the implementation of preventive measures. Conversely, regret could also increase errors due to concentration problems of the healthcare providers, but also because of a widespread use of a strategy of acceptance of medical errors [19], [25]. This strategy of acceptance, which is rarely used in the general population [26], could be dangerous for patient safety if it led to greater acceptance of preventable mistakes.

At the institutional level, regret could be one of the causes of absenteeism and turnover rate among nurses [27] since it sometimes led to sleep problems, taking sickness leave days or to definitive change in professional trajectories. Indeed, our sample of working healthcare providers by definition excluded all caregivers who quit their job and thus reported comments about turnover were probably partial and underrepresented.

Finally, one finding may impact on all three levels. While nurses talked more to colleagues of the same unit, physicians, on the contrary, avoided close colleagues. Indeed, physicians reported feeling that it was risky to talk to close colleagues because they could lose their credibility [28]. At the individual level, this barrier to physician peer support may block an effective coping strategy. At the patient care level, it could lead to fewer requests for clinical support [29] and thus suboptimal decisions and actions [30]. At the hospital level, it may lead to fewer incident reports [31].

### Strengths and limitations

This study used rich data provided by a diverse sample of informants on an important but underinvestigated topic. To minimize the risk of taking some observations for granted, the interviews were conducted by a non healthcare provider (i.e., a sociologist). However, to minimize the risk of missing important contextual information in the interviews, transcripts were reviewed by physicians, who also provided an accurate interpretation of the clinical situations.

As in all qualitative studies, our study sample was small and the generalizability of findings may be limited to people and settings with characteristics similar to those studied here. Furthermore, our sample was composed of volunteers, who may have experienced more regret or regret of stronger intensity than the general healthcare providers. This study examined regret and related coping strategies retrospectively. Thus, respondents' reports may be limited by recall bias. In addition, recent regrets often concern actions whereas older regrets are mostly regrets of inaction [32]. Since respondents mostly reported situations that occurred during their first years of clinical practice, the occurrence of regrets of inaction may be overestimated in this study. Moreover, some cognitive strategies such as reappraisal (analyses of what happened, and what went wrong) may have transformed the recall of the situation so that they no longer appear regret-inducing. These strategies would then not be reported.

### Conclusion

Since regret seems to be related to negative consequences such as sleep problems and turnover but also to positive consequences such as changes in practice to avoid further regret-inducing situation, it is important to learn more about regret regulation among healthcare providers. Further research is needed to explore regret prospectively and to determine whether training in regulation strategies could reduce negative consequences such as sleep problems, absenteeism, or turnover.

## Supporting Information

Box S1 Interview Guide.
(DOCX)

Box S2 Examples of regretted situations involving mistakes.
(DOCX)

Box S3 Examples of regretted situations not involving mistakes.
(DOCX)
