# Supplementary material for: Regrets Associated with Providing Healthcare: Qualitative Study of Experiences of Hospital-Based Physicians and Nurses
Source: PLoS One. 2011 Aug 2;6(8):e23138. doi: 10.1371/journal.pone.0023138 (PMC3149073; doi:10.1371/journal.pone.0023138)
Supplement: Box S1 — Interview Guide. (DOCX) [file pone.0023138.s001.docx]

Clinical decisions or actions may not always yield the best results. Indeed, patients’ conditions may even worsen whether or not the decision or action was the best possible choice. We would like you to talk about two situations that led to a regret, one in which you feel you may have made a mistake and one in which you did not. In both cases, you should report your most significant regret. For each regret:

Eliciting event

What was your most significant regret following a clinical decision or intervention?

Were any of your colleagues aware of the event?

Associated emotions and consequences

What emotions did you feel at the moment of the event?

What did you think at the moment of the event?

Some people feel regrets physically in their bodies, whereas others do not. How about you, what did it feel like?

On a scale ranging from 0 to 10 (0 being equal to no regret and 10 to very strong regret), what value would you ascribe to the intensity of this regret at the time of its first occurrence?

If this regret is still active and present, what is its intensity today?

Have thoughts in connection with this event kept you awake at night?

To what extent did this regret intrude in (e.g., prevented you from concentrating on) your private life? And in your professional life?

Regret coping strategies

How did you cope with this regret?

Have you changed your clinical practice in the wake of this event? If yes, how?

To what extent have you talked about it with colleague(s)? With your superiors? With your friends and family?
