# Supplementary material for: Regrets Associated with Providing Healthcare: Qualitative Study of Experiences of Hospital-Based Physicians and Nurses
Source: PLoS One. 2011 Aug 2;6(8):e23138. doi: 10.1371/journal.pone.0023138 (PMC3149073; doi:10.1371/journal.pone.0023138)
Supplement: Box S2 — Examples of regretted situations involving mistakes. (DOCX) [file pone.0023138.s002.docx]

Physicians

1. A house officer treating a patient with a metatarsal fracture gave non-steroidal anti-inflammatory drug to avoid deep venous thrombosis. Ten days later, the patient was readmitted for a massive pulmonary embolism. The patient responded well to treatment. The house officer later learned that he should have given anti-coagulants instead.

2. An obstetrician, thinking out loud, inadvertently informed the parents about the sex of their expected twins. Parents were shocked and told her that they did not want to know.

3. During a night shift, a young house officer in paediatrics admitted a child with a recent renal graft. The patient had a respiratory arrest related to a pulmonary oedema that the house officer had not detected. The patient was intubated and transferred to the ICU. No information on follow-up.

4. An anaesthetist in charge of the recovery room broke bad news to the spouse of a patient. While speaking, he realised that he was talking about the wrong patient. He apologised to the spouse and told her that her husband was doing well.

5. A physician provided anaesthesia to a 85-year-old woman with several co morbidities using a regimen appropriate for a healthy 20-year-old patient. The patient had a cardiac arrest, and was reanimated successfully without further consequences.

Nurses

6. A nurse started the infusion of chemotherapy on a patient without having received the results of the last blood tests and the physician’s authorisation. Blood tests later confirmed that the patient had no contraindication for this chemotherapy.

7. A nurse put the vaginal probe used to examine an HIV+ patient in a common cleaning tub for all probes. She immediately discarded all probes and phoned a colleague working with HIV patients. Her colleague told her that there was no significant risk of infection.

8. A nurse entered a room to administer insulin to a patient and called her name. One of the two patients present said yes and the other did not react, so the nurse gave the insulin dose to the first patient. Thirty minutes later, the nurse realised that she gave the insulin to the wrong patient. There were no consequences.

9. A nurse injected 10 times the dose of an analgesic drug to a new born in an ambulatory setting. The baby had to stay under observation for a night; there were no consequences for his health.

10. A nurse student was supposed to give an aerosol to a patient under continuous oxygen therapy. The aerosol had to be connected to the mask. She asked the patient, who seemed cognitively sound and had already done the operation under supervision, to do it herself, and then left the room. Twenty minutes later, she found the patient unconscious without her mask. The patient was sent to the ICU and died shortly thereafter.

11. A nurse in a psychiatric emergency ward was asked by a recently admitted well-known patient to look into her cell phone, get the number of her new companion and call him. The nurse did not do so, but nevertheless told the patient that she tried and did not manage to reach him.
