# Supplementary material for: Regrets Associated with Providing Healthcare: Qualitative Study of Experiences of Hospital-Based Physicians and Nurses
Source: PLoS One. 2011 Aug 2;6(8):e23138. doi: 10.1371/journal.pone.0023138 (PMC3149073; doi:10.1371/journal.pone.0023138)
Supplement: Box S3 — Examples of regretted situations not involving mistakes. (DOCX) [file pone.0023138.s003.docx]

Physicians

1. A house officer looked at a follow-up x-ray of a patient who had been hospitalised for pneumonia. He told his chief that he thought the pneumonia might have recurred. His chief told him to wait. At the visit the next day, the patient was much worse. Later that day, the patient went into septic shock, was transferred to the ICU, and died.

2. A drug user patient was treated for pneumonia without significant improvement. The house officer in charge wanted to perform additional tests but her chief disagreed. She regretted not standing up to her chief and wondered if the patient had side-effects due to delaying the more appropriate treatment.

3. A patient with a chronic disease did not take his treatment regularly, thus considerably reducing its effectiveness and potentially inducing resistance. Hoping to change his behaviour, a physician talked harshly to him. The patient broke contact.

4. A patient was admitted with a dissecting thoracic aorta aneurism and had to undergo urgent surgery. A senior house officer, who had not slept all night for personal reasons, did not report his tiredness and performed the surgery nevertheless. The surgery went well and the patient survived.

5. At the end of a long operation that went well, a patient was extubated and showed respiratory distress. The reintubation was difficult and the physician tried to perform a tracheotomy. He failed, which caused massive subcutaneous emphysema. The patient died.

6. An anaesthetist told a patient that she would perform the anaesthesia on her but finally was not able to do it herself. In the recovery room, the patient reproached her for not doing it.

7. A physician was sent by helicopter to rescue a patient in a mountain accident. Despite the physician’s efforts, the patient died.

8. A cardiac catheterization was performed on a patient with a massive pulmonary embolism. The patient could not be sedated during the procedure for medical reasons, and thus remained awake and in pain. The patient had a complication and died during the procedure.

Nurses

9. A nurse who was alone at night had to face several difficult situations at the same time: supporting a family after an expected death, attending to a patient in agranulocytosis who fell and broke her nose, a patient with ileus and acute chest pain, and a COPD patient in respiratory distress. She regretted not being able to deal to her satisfaction with all these situations because she was too stressed.

10. A patient with respiratory distress was transferred from the emergency room to a medical ward for palliative care. The patient died less than two hours later and was found by his brother with his mask between his legs. The nurse regretted that she did not provide comfort care immediately and that the patient was found by a family member.

11. A patient in a medical ward, who was supposed to receive only palliative care, was still examined regularly. The nurse had to take blood samples every two hours and temperature every 30 minutes. The patient died one hour after the last blood tests.

12. A patient was supposed to receive a permanent catheter. The nurse received information that his platelet count was too low but did not reprogram the procedure. The patient had to come back at a later date, and the nurse unknowingly transfused him with non-conform platelets that caused a massive shock. The patient survived. The nurse regretted not seeing that his platelets count was too low before his visit because he would then have been transfused with another batch of platelets.

13. A patient who had been hospitalized for three weeks finally died in acute respiratory distress. Thinking the patient would survive, the nurse did not call the family, even though she knew that the family wanted to be present at the end.

14. A nurse was attending to a 40-year-old patient with terminal cancer in much pain. The nurse thought that she would probably die during the night, but felt incapable of seeing her because it was “emotionally unbearable”. She asked a colleague to go and see her in her place, but her colleague answered that she was too busy. The patient died alone during the night.
